# More is not always merrier: Enhanced atmospheric CO_2_ reduces nutritional seed quality in oak trees

**DOI:** 10.1093/plphys/kiaf491

**Published:** 2025-10-05

**Authors:** Gunjan Sharma

**Affiliations:** Assistant Features Editor, Plant Physiology, American Society of Plant Biologists; School of Biosciences, University of Birmingham, Edgbaston B15 2TT, UK

Carbon dioxide (CO_2_) is obligatory for photosynthesis with an abundance in the environment. However, a gradual increase in atmospheric CO_2_ has led to its greater availability as a substrate for photosynthesis in plants. Plants cope with enhanced CO_2_ (eCO_2_) by optimizing the key enzyme ribulose-1, 5-bisphosphate carboxylase/oxygenase (RUBISCO) and other photosynthetic proteins (Ancin et al. 2024), thereby increasing photosynthesis and growth of C3 annual plants—known as the CO_2_ fertilization effect. eCO_2_ treatment also reduces photorespiration, impairing nitrogen assimilation. Despite increased growth, decreased protein content in leaves and seeds is a negative consequence of eCO_2_ (Shi and Bloom 2021).

Seeds and nuts are an integral part of the human diet, and forest tree nuts are key in maintaining forest food webs (Ickowitz et al. 2022). Development of seeds requires de novo synthesis and mobilization of essential carbohydrates, carbon metabolites, and amino acids from surrounding aerial tissues and micronutrients from the soil (Pirredda et al. 2024). Artificially imposed eCO_2_ levels in free-air carbon enrichment experiments have predicted the reduction in nutritional seed quality. Poor bioavailability and absorption of micronutrients such zinc and iron through increased potent inhibitors such as phytates, myoinositols, hexaphosphate, and polyphenols were observed (Gibson et al. 2018; Bouain et al. 2022). Interestingly, eCO_2_ levels increased mature viable seed production in pine trees without affecting seed quality (Way et al. 2011). However, a reduction in herbivores over 9 yr suggested poor seed nutritional quality (Stilling and Cornelissen 2007). These reports demonstrate that the effects of eCO_2_ on seed and fruit nutritional quality are not easy to predict and underlying molecular mechanisms in perennial trees are yet unknown.

In a recently published article in *Plant Physiology*, Karpinska et al. (2025) assessed the effect of eCO_2_ on the nutritional quality of acorns. The acorns were produced by oak trees exposed to eCO_2_ supply for up to 8 yr and showed altered protein profiles and phytate levels. These observations pinpointed molecular components and signaling pathways responsible for redirecting metabolism by shifting carbon flow.

The authors set out to assess the long-term effect of eCO_2_ on oak trees at the BIFoR FACE facility (Figure A). The acorns from mature old oak trees growing in eCO_2_ conditions were collected in Years 7 and 8. As reported earlier, eCO_2_ increased the acorn size as compared with ambient CO_2_ (aCO_2_)–grown trees. However, acorns with uniform size and weight were chosen for analysis to avoid any variables arising from size variation. Despite the larger acorn size, no difference in seed germination rate or germination efficiency was observed (Figure B).

**Figure.**
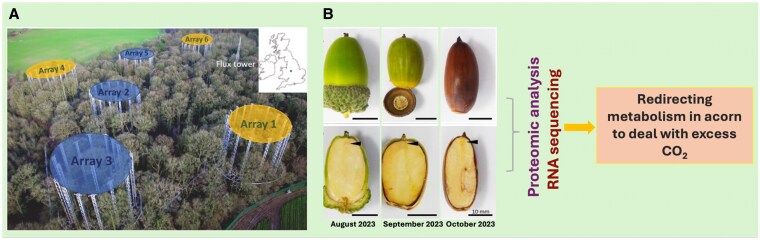
Experimental setup and representative images of the range of acorn developmental stages harvested from eCO_2_-flushed oak trees. **A)** Schematic of the BIFoR-FACE site in Staffordshire, United Kingdom. Fumigated arrays flushing increased CO_2_ to oak trees are represented as Array 1, 4 and 6, whille ambient are Array 2, 3 and 5. Panel A reprinted from *Rumeau et al. (2024)*  https://doi.org/10.1016/j.soilbio.2024.109537 which is licenced under CC-BY-4.0 https://creativecommons.org/licenses/by/4.0/. **B)** Oak acorns, whole and sectioned, were used in the study as collected from eCO_2_-flushed oak trees. These acorns were subjected to proteomic and RNA sequencing analyses to identify global changes redirecting their metabolism to deal with the eCO_2_ fertilization effect. Adapted from Karpinska et al. (2025).

Larger seed size prompted the authors to examine total protein levels in acorns produced from eCO_2_- and aCO_2_-grown trees. eCO_2_ decreased the total protein content in acorns as compared with aCO_2_-grown trees. An increase in phosphorus and phytate content was observed. A cost-effective and wider-coverage label-free proteomic analysis revealed that 335 proteins consistently present in all sample types. Of these 335 proteins, 32 were underrepresented in eCO_2_ while 11 were more abundant in comparison with aCO_2_. Notably, these less abundant proteins were key enzymes catalyzing glycolysis, gluconeogenesis, tricarboxylic acid cycle, and synthesis of secondary compounds, suggesting a slowdown in carbon reduction. Conversely, proteins involved in carbon oxidation pathways such as those for protein glycosylation were more abundant. The differential protein accumulation in acorns produced from eCO_2_-exposed trees suggested a transition from carbon reduction to oxidation to deal with an excess of CO_2_, supporting plant defense.

To explore global transcriptomic changes underlying oak seed nutritional quality and nutrient mobilization during eCO_2_, the authors performed RNA sequencing on the same subset of acorns used for seed protein profiling. RNA sequencing revealed 154 transcripts being abundant in eCO_2_ acorns as compared with aCO_2_, while 54 were less abundant. One of the key upregulated genes proved to be a trehalose-6-phostase regulating sugar metabolism and signaling, linking carbon status of the plant with growth and development (Fichtner and Lunn 2021). Other transcripts upregulated in eCO_2_ acorns were iron starvation response markers. Although no changes in micronutrient concentrations were observed in acorns produced from eCO_2_- or aCO_2_-flushed trees, increased levels of iron chelator phytates were observed, suggesting an effect on iron accumulation and bioavailability. Interestingly, downregulated genes were mostly found to be involved in regulation of cell signaling proteins, heat shock factors, and poly (ADP-ribose) polymerase.

In conclusion, Karpinska et al. (2025) demonstrated that eCO_2_ availability alters acorn seed quality in oak trees. The acorns exhibited reduced protein content and adapted to eCO_2_ by redirecting their metabolism to accumulate more phytate content and fewer seed proteins with less bioavailability of an essential micronutrient (i.e. iron). The reduced nutritional seed quality in eCO_2_ might be a reason for the reduced loss of acorns to herbivores in a previous study. The authors have generated robust resources for future studies focused on trees such as oaks for investigating the implications of an eCO_2_ fertilization effect.

## Data Availability

No data were generated or analyzed in this study.
